# Predicting cerebral palsy and 18-month neurodevelopmental outcome in infants with presumed hypoxic ischaemic encephalopathy: role of general movements assessment and early neurological examination

**DOI:** 10.3389/fped.2025.1638584

**Published:** 2025-10-03

**Authors:** Gugulabatembunamahlubi T. J. Kali, Jacomina C. F. du Preez, Jeanetta I. van Zyl, Marlette Burger, Hillary Katsabola, Michael S. Pepper

**Affiliations:** ^1^ Division of Neonatal Medicine, Department of Paediatrics and Child Health, Faculty of Medicine and Health Sciences, Stellenbosch University, Cape Town, South Africa; ^2^ Department of Paediatrics and Child Health, Tygerberg Hospital, Cape Town, South Africa; ^3^ Division of Physiotherapy, Department of Health and Rehabilitation Sciences, Faculty of Medicine and Health Sciences, Stellenbosch University, Cape Town, South Africa; ^4^ Division of Epidemiology and Biostatistics, Faculty of Health Sciences, Stellenbosch University, Cape Town, South Africa; ^5^ Department of Medical Immunology, Faculty of Health Sciences, Institute for Cellular and Molecular Medicine, and SAMRC Extramural Unit for Stem Cell Research and Therapy, University of Pretoria, Pretoria, South Africa

**Keywords:** hypoxic–ischaemic encephalopathy, cerebral palsy prediction, general movements assessment, Motor Optimality Score—Revised, early neurological examination

## Abstract

**Introduction:**

General movements assessment (GMA), including the Motor Optimality Score—Revised (MOS-R) and the Hammersmith Infant Neurological Examination (HINE), has been shown in different settings to predict cerebral palsy (CP) and delayed neurodevelopment with high accuracy. However, their combined predictive ability has not been fully evaluated in infants with presumed hypoxic–ischaemic encephalopathy (HIE).

**Objective:**

This study aimed to assess the predictive ability of combined GMA, MOS-R, and HINE at 3 months in term or near-term infants diagnosed with presumed HIE, for neurodevelopmental outcome at 18 months.

**Methods:**

A cohort of presumed HIE infants treated with therapeutic hypothermia (TH) underwent GMA, MOS-R, and HINE at 12–15 weeks, and neurodevelopmental assessments using the Bayley Scales of Infant and Toddler Development Third Edition (BSID-III) at 9–12 and at 18–24 months of age. Combined early assessments were analysed for their predictive ability across different domains on the BSID-III.

**Results:**

Twenty-four infants were included; 7 (29%) had both 12-month and 18-month BSID-III assessments, 12 (50%) were seen only at 12 months, and 5 (21%) only at 18 months. Two infants with absent fidgety movements (FMs) and poor motor repertoire were later diagnosed with CP or showed delays in two domains on the BSID-III assessment at 18 months. While most infants had some abnormality in the MOS-R categories, only absent FMs and abnormal finger variability showed some association with the 18-month BSID-III assessment on univariate analysis. Of the four infants classified as at risk for CP on the HINE at 3 months, two had some motor abnormalities at 18 months. Combining the GMA, MOS-R, and HINE had high sensitivity and negative predictive value (100%), but low specificity (0–17.6%) and positive predictive value (6.2%–25%) for the BSID-III outcome.

**Conclusion:**

Combining GMA, MOS-R, and HINE was highly sensitive in this cohort, but had low specificity. This may lead to overdiagnosis, but it may be a useful screening tool for identifying typically developing infants who do not need intensive follow-up.

## Introduction

Presumed hypoxic–ischaemic encephalopathy (HIE) remains a significant cause of neonatal mortality and long-term neurodevelopmental impairment, particularly in low- and middle-income countries (LMIC) (1). Despite advancements in neonatal care, including the implementation of therapeutic hypothermia (TH), a substantial proportion of infants with HIE continue to experience adverse outcomes, such as high mortality, cerebral palsy (CP) with associated morbidities such as seizures, and cognitive delays (2, 3).

Although TH has improved outcomes, a significant proportion (23%) of infants still develop CP. Early identification of those at risk for CP is therefore crucial for initiating timely interventions that could potentially modify developmental trajectories, prevent associated complications, and lighten caregiver load. Prechtl's General Movements Assessment (GMA) has emerged as a reliable, non-invasive tool for early detection of neurological dysfunction (4). Specifically, the absence of fidgety movements (FMs) at 3–5 months post-term age has been strongly associated with later diagnosis of CP or significant developmental delay (5–7). The Hammersmith Infant Neurological Examination (HINE), a structured neurological assessment, is predictive of motor outcomes when administered between 2 and 24 months of age and has been shown to complement the predictive ability of GMA for CP (7, 8). Recent studies have highlighted the enhanced predictive value of combining GMA and HINE assessments to improve early diagnostic accuracy for CP in cooled and uncooled HIE infants (8, 9). Furthermore, the Motor Optimality Score—Revised (MOS-R), a quantitative extension of the concurrent motor repertoire of GMA, has demonstrated improved specificity in predicting motor impairments at 12 and 24 months in high-risk populations such as term infants with HIE (3). In this scoring system, the FMs and their concurrent motor repertoire, namely, the MOS-R, are assessed and scored as described by Einspieler et al. in 2019 (10).

The prognostic utility of GMA and HINE is further supported by their correlation with neuroimaging findings. Combining neonatal MRI with GMA has been shown to predict neurodevelopmental outcomes with high accuracy, offering a feasible approach in some resource-limited settings (11, 12). Moreover, the application of these assessments in low- and middle-income countries has demonstrated their effectiveness in early detection of CP and facilitating the possibility of timely intervention strategies during the period of neuroplasticity (5, 6, 9).

This paper aims to evaluate the predictive validity of MOS-R and early neurological examinations (particularly HINE) in forecasting CP and neurodevelopmental outcomes at 18 months in infants with presumed HIE treated with TH in our setting, where MRI is not routinely available, and follow-up is challenging.

## Methods

### Design and setting

This is a sub-study of an ongoing study, the Neonatal Encephalopathy with Suspected Hypoxic Ischaemic Encephalopathy (NESHIE) study (HREC reference no: N18/03/041_RECIP_UP-481/2017), an observational study of infants with moderate to severe encephalopathy treated with TH at Tygerberg Hospital (TBH) in Cape Town, South Africa, between June 2019 and August 2023. This hospital delivers approximately 8,000 babies annually, has 12 neonatal critical care beds, and provides tertiary care to half of the neonates in the Western Cape province.

TH is provided in the neonatal intensive care unit (NICU), and infants with moderate–severe presumed HIE who have been cooled are routinely followed up as outpatients at the high-risk clinic (HRC) of TBH.

### Participants

Infants were included if they met the following criteria: had moderate–severe presumed HIE and had received TH; had 5 min video recordings of general movements (GMs) at 12–15 weeks; had neurological examinations performed using the HINE at 12–15 weeks corrected age; and had neurodevelopmental assessments using the Bayley Scales of Infant and Toddler Development Third Edition (BSID-III) at either 9–12 or 18–24 months corrected age.

Infants who did not have both early assessments at 12–15 weeks and at least one of the later neurodevelopmental assessments (BSID-III at 12 months or 18 months) were excluded.

### GMA

Video recordings of spontaneous infant movements were captured between 12 and 15 weeks corrected age following established GMA protocols as described by Einspieler (10). Each recording session lasted 5 min with infants positioned supine during alert, calm states between feeding times. When infants became distressed (hiccupping, fussing, or crying), recordings were temporarily halted to allow caregivers to settle the infant before continuing.

Movement assessment was conducted by three certified GMA evaluators with advanced training from the General Movements Trust. Videos were scored independently, with discrepancies resolved through collaborative review to establish consensus ratings. Assessment was performed with evaluators masked to clinical history and developmental outcomes, except for one assessor (JvZ) who had access to this information. Each assessor scored the videos based on specific components of the MOS-R, including the presence and quality of FMs, postural patterns, finger variability, movement character, total MOS-R, and a consensus decision was then reached for each video.

### Neurological assessments

All the patients were serially assessed at a neurodevelopmental clinic as part of the follow-up protocol for high-risk infants in the treating hospital. Normal outcome and CP diagnosis were clinically made based on a neurological examination of tone, reflexes, posture, and gross and fine motor developmental ability. These assessments included neurological examinations using the HINE at 3 months (12–15 weeks) corrected age and neurodevelopmental assessments using the BSID-III at 12 and 18 months. All assessments were performed by a single experienced developmental specialist and, apart from the BSID-III assessments which were done for study purposes, were routinely done in the HRC.

A score of <57 on the HINE was regarded as at risk for CP.

The BSID-III is a widely accepted, norm-referenced assessment instrument. International organisations, including the World Bank Group, have endorsed it as the preferred measurement tool for developmental assessment in infants and toddlers within low- and middle-income countries (13). While African-specific normative data for the BSID-III remain unavailable in the published literature, validation studies have been conducted among healthy infants between 2 and 13 months of age in South Africa, demonstrating comparable performance scores to the original US normative sample (14). Another study involving healthy urban toddlers in South Africa (mean age 19.4 months) found developmental scores that fell somewhat below the 50th percentile of the American BSID-III reference standards, although these results remained within normal developmental ranges across the various assessed areas (15). BSID-III scores were allocated for three domains of development (motor, communication, and cognitive) as follows: ≥85 competent, 70–84 at risk, and <70 delayed.

### Statistical analysis

Descriptive statistics were used to summarise baseline characteristics and developmental outcomes at 12 and 24 months of age. Continuous variables were evaluated for normality using the Shapiro–Wilk test, with normally distributed variables summarised using means and standard deviations (SD) and non-normally distributed variables using medians and ranges, while categorical variables were presented as counts and percentages.

To assess associations between early clinical findings and neurodevelopmental outcomes at 12 and 18 months, Fisher's exact test was used for categorical variables, appropriate for small sample sizes and sparse cell counts. Continuous variables were compared using Student's *t*-test for independent samples under the assumption of approximate normality (16). *p*<0.05 was considered significant.

Diagnostic performance of combined clinical variables was evaluated for multiple neurodevelopmental outcomes on the BSID-III, namely, cognitive, communication, and motor domains at 12 and 18 months. Metrics including sensitivity, specificity, positive predictive value (PPV), negative predictive value (NPV), and prevalence were calculated to quantify classification accuracy. These metrics were calculated using standard 2 × 2 contingency tables.

Statistical analyses were performed using R version 4.4.3 (2025-02-28) “Trophy Case.”

## Results

**Figure 1:**
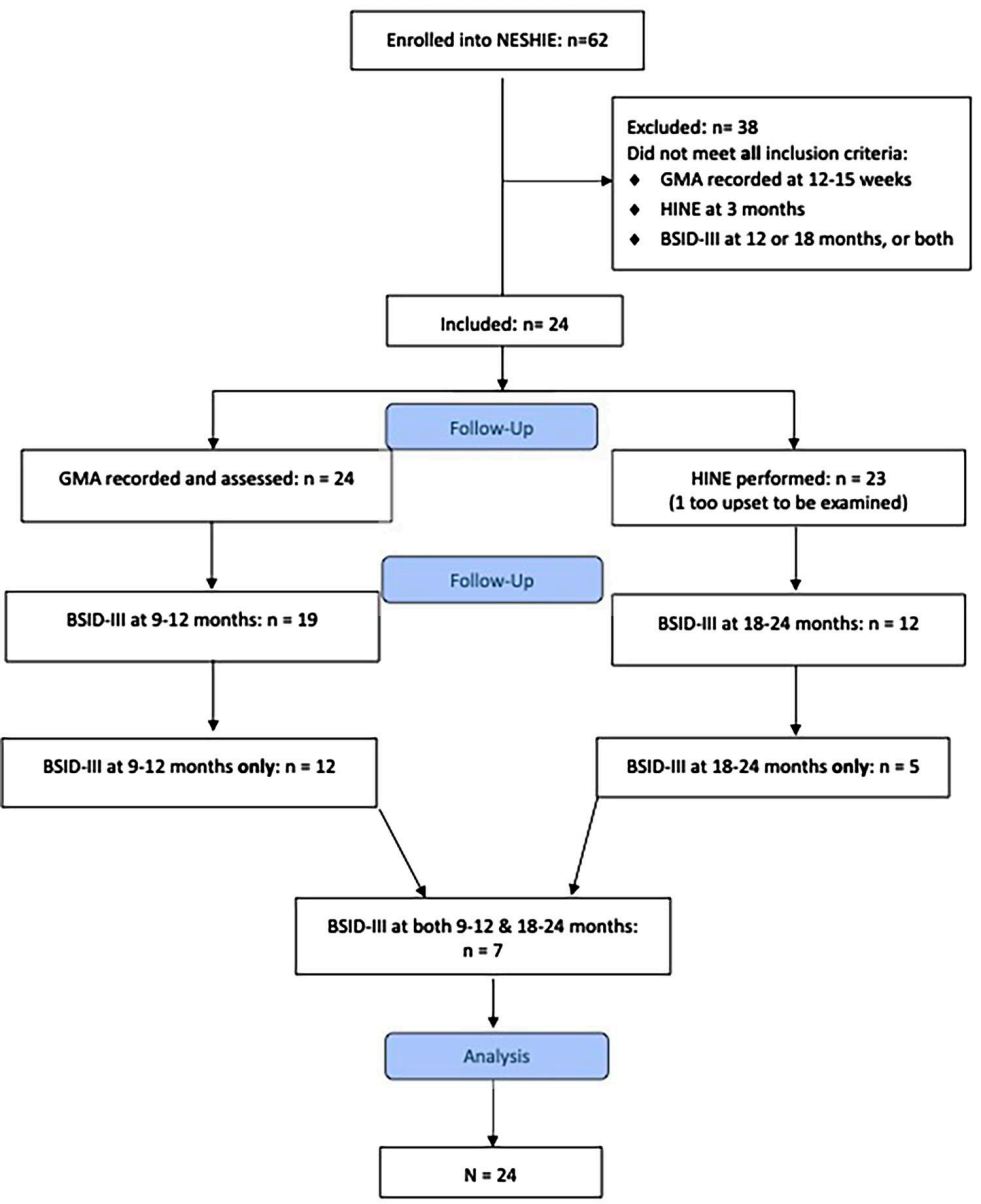
Study enrolment process.

Twenty-four infants met the inclusion criteria (see Figure 1). Baseline characteristics of the cohort are described in Table 1. All 24 infants had completed MOS-R, and 23 infants had the HINE performed at 3 months (12–15 weeks). One infant had the HINE deferred as he was too upset to be examined. Nineteen infants had BSID-III assessments at 9–12 months of age, 12 were assessed at 18–24 months, and 7 were assessed at both time points.

**Table 1:** Baseline perinatal characteristics of cohort.

Clinical variables	*N* = 24
Maternal and delivery data	
Maternal age, median (range), years	23 (18–37)
Primiparity, *n* (%)	18 (75.0)
HIV positive, *n* (%)	1 (4.2)
Pregnancy complications, *n* (%)	15 (62.5)
Sentinel event, *n* (%)	6 (25.0)
Emergency CS, *n* (%)	9 (37.5)
Assisted delivery, *n* (%)	5 (20.8)
Infant data	
Inborn, *n* (%)	7 (29.2)
Male, *n* (%)	12 (50.0)
Gestational age, median (range), weeks	39 (36–41)
Birthweight, median (range), g	3,115 (2,100–4,925)
HC at birth, median (range), cm	35 (33–40)
Apgar 1 min, median (range),	3 (0–6)
Apgar 5 min, median (range)	5 (2–7)
Apgar 10 min, median (range)	6 (2–9)
pH within first hour <7, *n* (%)	18 (75.0)
pH within first hour, median (range)	6.99 (6.69–7.22)

### Clinical assessments

The neonatal clinical conditions and findings from assessments of MOS-R and HINE at 12–15-week (3 months) follow-up are described in Table 2.

**Table 2:** Clinical assessments of infants.

Clinical variables	*N* (%)
Neonatal period	
HIE 2	21 (87.5)
HIE 3	3 (12.5)
48 h aEEG	
CNV	11 (45.8)
DC	3 (12.5)
CLV/BS/FT	10 (41.7)
3 months	
HINE	
Normal	19 (82.6)
At risk of CP	4 (17.4)
General movements assessment	
Fidgety movements	
Present	22 (91.7)
Absent	2 (8.3)
MOS-R category (total score: 28)	
Optimal (25–28)	5 (20.8)
Mildly reduced (20–24)	17 (70.8)
Moderately reduced (9–19)	2 (8.3)
Severely reduced (≤8)	0 (0)
Postural pattern	
Optimal symmetry	5 (20.8)
Suboptimal symmetry	13 (54.2)
Decreased symmetry	6 (25.0)
Finger variability	
Good variability	19 (79.2)
Asymmetrical	3 (12.5)
Not good variability and asymmetrical	2 (8.3)
Movement character	
Smooth and fluent	8 (33.3)
Abnormal, not cramped synchronised	16 (66.7)
Cramped synchronised	0 (0)

HIE, hypoxic ischaemic encephalopathy; aEEG, amplitude integrated electroencephalography; CNV, continuous normal voltage; DC, discontinuous; CLV, continuous low voltage; BS, burst suppressed; FT, flat trace; HINE, Hammersmith Infant Neurological Examination; CP, cerebral palsy; MOS-R, Motor Optimality Score—Revised.

The majority (87.5%) of infants were classified as moderate encephalopathy on admission. Over half (54.2%) still had abnormal amplitude integrated electroencephalograph (aEEG) pattern at 48 h, with 42% being severely abnormal.

All infants except one had some abnormality in the subcategories of the MOS-R. Two had absent FMs, and none had cramped synchronised movements. The characteristics of the two infants with absent FMs are depicted in Table 3.

**Table 3:** Characteristics of infants with absent fidgety movements.

Clinical variables	Infant 1	Infant 2
Maternal characteristics		
Maternal age, years	25	23
Parity, *n*	1	1
Delivery mode	NVD	Vacuum
Infant characteristics		
Birth site	Outborn	Outborn
Gestational age, weeks	36	40
Birthweight, grams	2,610	3,210
Head circumference, cm	34	34
Sex	Male	Male
Resuscitation	CPR	CPR
Apgar 5 min	3	5
HIE grade	2 (Thompson 9)	2 (Thompson 9)
Seizures on admission	No	Yes (aEEG)
aEEG 48 h	DC	BS
General movements assessments		
Fidgety movements	Absent	Absent
MOS-R category	Moderately reduced	Moderately reduced
Postural pattern	Optimal symmetry	Optimal symmetry
Finger variability	Asymmetrical	Asymmetrical
Movement character	Jerky	Monotonous
HINE		
HINE at 3 months	ND	At risk of CP (<57)
BSID-III at 18 months		
Motor	70–84	<70
Communication	70–84	70–84
Cognitive	≥85	<70
Latest known clinical outcome		
Latest known clinical outcome	Spastic diplegia; gross and fine motor and speech delay at 3.5 years	Moderate spastic quadriplegia with dystonia at 2 years

MOS-R, Motor Optimality Score—Revised; HINE, Hammersmith Infant Neurological Examination; CP, cerebral palsy; BSID-III, Bayley Scales of Infant and Toddler Development Third Edition; NVD, normal vaginal delivery; CPR, cardiopulmonary resuscitation.

Of the four infants who were classified as at risk for CP on the HINE at 3 months, one had delays on all domains of the BSID-III at 18 months and was later diagnosed with spastic quadriplegic CP with dystonia. Another infant was classified as at risk in the motor category of the BSID-III at 18 months, walked after 18 months, and demonstrated mild motor delays at 3 years of age. The other two infants had normal motor outcomes at either the 12- or 18-month assessment.

The one infant who had no abnormal findings on the MOS-R and had a normal score on the HINE at 3 months was competent on all three domains (motor, cognitive, communication) of the BSID-III at 18 months of age.

### Prediction of outcome

Associations between early assessments and BSID-III outcomes at 12 and 18 months are shown in Tables 4 and 5. On univariate analysis, absent FMs and abnormal finger variability showed a significant association with being classified as at risk or delayed on BSID-III assessment at 18 months (Table 5), but no association with the 12-month BSID-III assessments was found (Table 4).

**Table 4:** Association between early findings and outcomes at 12 months.

Clinical variables	At risk (*N* = 4) (BSID-III <70–84)	Not at risk (*N* = 15) (BSID-III ≥85)	*P*-value
Age 3 month visit			
Mean (SD)	13.8 (0.957)	13.3 (0.976)	0.477
Median [min, max]	13.5 [13.0, 15.0]	13.0 [12.0, 15.0]	
HIE grade at admission, *n* (%)			
Moderate	2 (50.0)	14 (93.3)	0.097
Severe	2 (50.0)	1 (6.7)	
48 h aEEG, *n* (%)			
CNV	3 (75.0)	7 (46.7)	0.756
DC	0 (0)	2 (13.3)	
CLV/BS/FT	1 (25.0)	6 (40.0)	
Fidgety movements, *n* (%)			
Present	3 (75.0)	15 (100)	0.211
Absent	1 (25.0)	0 (0)	
MOS-R category, *n* (%)			
Optimal	0 (0)	4 (26.7)	0.225
Mildly reduced	3 (75.0)	11 (73.3)	
Moderately reduced	1 (25.0)	0 (0)	
Postural pattern, *n* (%)			
Optimal symmetry	1 (25.0)	2 (13.3)	0.432
Suboptimal symmetry	3 (75.0)	8 (53.3)	
Decreased symmetry	0 (0)	5 (33.3)	
Finger variability, *n* (%)			
Good variability	2 (50.0)	13 (86.7)	0.0699
Asymmetrical	2 (50.0)	0 (0)	
Not good variability and asymmetrical	0 (0)	2 (13.3)	
Movement character, *n* (%)			
Smooth and fluent	0 (0)	6 (40.0)	0.255
Abnormal, not cramped synchronised	4 (100)	9 (60.0)	
HINE, *n* (%)			
Normal	2 (50.0)	13 (86.7)	0.178
At risk of CP	2 (50.0)	2 (13.3)	

BSID-III, Bayley Scales of Infant and Toddler Development Third Edition; HIE, hypoxic–ischaemic encephalopathy; aEEG, amplitude integrated electroencephalography; CNV, continuous normal voltage; DC, discontinuous; CLV, continuous low voltage; BS, burst suppressed; FT, flat trace; MOS-R, Motor Optimality Score—Revised; HINE, Hammersmith Infant Neurological Examination; CP, cerebral palsy.

**Table 5:** Association between early findings and outcomes at 18 months.

Clinical variables	At risk (*N* = 3) (BSID-III <70–84)	Not at risk (*N* = 10) (BSID-III ≥85)	*P*-value
Age 3 month visit			
Mean (SD)	13.0 (0)	13.3 (1.16)	0.434
Median [min, max]	13.0 [13.0, 13.0]	13.0 [12.0, 15.0]	
HIE grade at admission, *n* (%)			
Moderate	3 (100)	10 (100)	1
Severe	0 (0)	0 (0)	
48 h aEEG, *n* (%)			
CNV	1 (33.3)	4 (40.0)	0.738
DC	1 (33.3)	1 (10.0)	
CLV/BS/FT	1 (33.3)	5 (50.0)	
Fidgety movements, *n* (%)			
Present	1 (33.3)	10 (100)	**0**.**0385**
Absent	2 (66.7)	0 (0)	
MOS-R category, *n* (%)			
Optimal	0 (0)	2 (20.0)	0.0769
Mildly reduced	1 (33.3)	8 (80.0)	
Moderately reduced	2 (66.7)	0 (0)	
Postural pattern, *n* (%)			
Optimal symmetry	2 (66.7)	3 (30.0)	0.738
Suboptimal symmetry	1 (33.3)	4 (40.0)	
Decreased symmetry	0 (0)	3 (30.0)	
Finger variability, *n* (%)			
Good variability	1 (33.3)	9 (90.0)	**0**.**0385**
Asymmetrical	2 (66.7)	0 (0)	
Not good variability and asymmetrical	0 (0)	1 (10.0)	
Movement character, *n* (%)			
Smooth and fluent	0 (0)	4 (40.0)	0.497
Abnormal, not cramped synchronised	3 (100)	6 (60.0)	
HINE, *n* (%)			
Normal	1 (50.0)	8 (80.0)	0.455
At risk of CP	1 (50.0)	2 (20.0)	

BSID-III, Bayley Scales of Infant and Toddler Development Third Edition; HIE, hypoxic–ischaemic encephalopathy; aEEG, amplitude integrated electroencephalography; CNV, continuous normal voltage; DC, discontinuous; CLV, continuous low voltage; BS, burst suppressed; FT, flat trace; MOS-R, Motor Optimality Score—Revised; HINE, Hammersmith Infant Neurological Examination; CP, cerebral palsy.

Bold indicate *P* values that were significant.

The predictive abilities of combined total MOS-R, FMs, finger variability, and HINE (Combined variables 1); and of combined total MOS-R, FMs, finger variability, HINE, HIE grade, and 48 h aEEG severity (Combined variables 2) are depicted in Table 6.

**Table 6:** Predictive abilities of combined GMA and clinical parameters.

Predictors	BSID-III outcome	Sensitivity %	Specificity %	PPV %	NPV %	Prevalence %
12 months						
Combined variables 1	Cognitive category	100	16.7	6.2	100	5.3
Combined variables 1	Communication category	100	17.6	12.5	100	10.5
Combined variables 1	Motor category	100	17.6	12.5	100	10.5
Combined variables 2	Cognitive category	100	16.7	6.2	100	5.3
Combined variables 2	Communication category	100	17.6	12.5	100	10.5
Combined variables 2	Motor category	100	17.6	12.5	100	10.5
18 months						
Combined variables 1	Cognitive category	100	0	8.3	NA	8.3
Combined variables 1	Communication category	100	0	16.7	NA	16.7
Combined variables 1	Motor category	100	0	25	NA	25
Combined variables 2	Cognitive category	100	0	8.3	NA	8.3
Combined variables 2	Communication category	100	0	16.7	NA	16.7
Combined variables 2	Motor category	100	0	25	NA	25

BSID-III, Bayley Scales of Infant and Toddler Development Third Edition; PPV, positive predictive value; NPV, negative predictive value.

Combined variables 1: FMs, MOS-R total score, finger variability, HINE.

Combined variables 2: FMs, MOS-R total score, finger variability, HINE, HIE, aEEG-48h.

## Discussion

We describe a cohort of cooled infants with moderate–severe presumed HIE who had MOS-R and HINE performed at 3 months, and at least one BSID-III assessment at 12 or 18 months of age.

The majority of infants had some abnormalities in the subcategories of the MOS-R. This is similar to a US study which found that moderate–severe HIE infants were most likely to have abnormal GMA at both term age and at 12 to <18 weeks corrected age (17). Most infants in the current study (87.5%) had moderate presumed HIE. We did not analyse MOS-R subcategory scores in relation to HIE severity to determine whether scores declined with increasing HIE severity, as demonstrated by Alkan et al. (18). Only two (8.3%) infants had absent FMs, and none had cramped synchronised movements, the other specific type of abnormal GM pattern that has been shown to predict CP, at 3 months of age (4).

Only four (16.7%) infants had abnormal HINE scores at 3 months. We did not find a significant association between early HINE assessments and BSID-III outcome at either 12 or 18 months. Our findings may align with those of Romeo et al. (19), who reported better overall HINE scores than a similar cohort from the pre-TH era. Although the HINE in this study was done at a later age than in our study, the authors suggested that the better HINE scores may reflect less cerebral injury in cooled infants. They also showed that only the infants with the lowest scores in the suboptimal range had severe motor impairment, indicating that perhaps the thresholds used for prediction pre-TH may need to be revised for cooled infants. These findings are consistent with what was reported by Ferrari et al. (20), where severely abnormal GMs were reduced after cooling, and there was a weaker association between absent FMs and the development of CP. These authors suggest that methods used to predict outcomes in the pre-cooling era may not be as reliable. One infant with an at-risk HINE score was delayed in all domains of the BSID-III at 18 months of age, consistent with the study by Romeo et al. (21) showing that HINE can predict delays in domains other than motor.

We found that combining MOS-R subcategories and HINE had perfect sensitivity (100%) and NPV (100%) for predicting adverse outcome in the motor, communication, and cognitive scales on BSID-III at 12 months of age and 100% sensitivity for predicting 18-month outcome in this cohort. Combining MOS-R, HINE, HIE grade, and 48 h aEEG had similar predictive ability. However, specificity and PPV were very low for both combinations. Specificity was lowest for the 18-month outcome, despite increasing prevalence of motor and communication impairments between 12 and 18 months (see Table 6). These findings are in contrast to those reported from both LMIC and high-income countries, where a combination of GMA (without MOS-R) and HINE showed high sensitivity and specificity for predicting CP (8, 9). These findings suggest that these examinations may serve as valuable screening tools, particularly in low-resource settings where MRI is not readily available. In such contexts, bedside examination findings could offer reassurance to clinicians and parents that the infant is unlikely to develop CP or significant impairments. This, in turn, could support more informed referral decisions and help allocate limited resources to the patients where they are most needed.

The need for accurate predictors of outcome, concurrent with the advances in management of HIE, has been advocated by Cizmeci et al. (22) in their review of neuroprognostication in HIE. They recommend the use of multiple tools to predict outcomes and facilitate early intervention and support families. Marlow et al. (23) noted that while follow-up results from three large TH trials showed that the beneficial effects appeared to persist until school age, the trials were not powered to detect these outcomes. They suggest that there is a need for longitudinal studies in the TH era to assess the impact of neonatal encephalopathy on cognitive, educational, and behavioural outcomes, as well as on families. Sensitive screening tools would assist in identifying infants with evolving problems that may benefit from closer follow-up and access to targeted interventions.

Different studies have shown that a combination of factors may be useful in predicting infants at risk of developing CP. A study of high-risk Italian infants found that CP could be predicted with >97% accuracy by combining neuroimaging, GMA, and HINE (24), while another Italian study by Lugli et al. (25) on cooled and uncooled infants with encephalopathy found that polygraphic EEG at 48 h and abnormal FMs had the best predictive ability for severe neurodevelopmental impairment at 2 years. Conversely, Glass et al. (12) showed that a normal neonatal MRI and GMA at 3 months in high-risk infants in the USA predicted a low risk of developing moderate/severe CP. These tools may assist in directing resources where long-term follow-up is challenging, provided they are available or readily accessible. A study by Aker et al. (11) in a similar LMIC showed that neonatal MRI and GMA at 10–15 weeks predicted outcome with similarly high accuracy in infants with HIE. This suggests that in settings where access to MRI is limited, GMA alone may be a feasible, low-cost predictive tool. GMA with MOS-R has also been shown to assist with localising injury in infants with perinatal arterial stroke and predicting unilateral CP, thereby enabling individualised targeted intervention (26).

As we do not have ready access to an MRI, we attempted to see whether predictive ability would be improved by combining subcategories of the MOS-R with early clinical findings (HIE, 48 h aEEG). However, this did not improve the specificity or PPV.

Several factors may explain the low specificity despite high sensitivity in our study.

The sample size was small, and there was a relatively low prevalence (5%–25%) of the adverse outcomes. TH may alter recovery and the developmental trajectory by enhancing neuroplasticity, so that some infants with early mild or non-specific signs may go on to recover with time, as suggested by Ferrari et al. (20). The specificity might have been improved by increasing the frequency of examinations, e.g., repeating the HINE at 6 months. However, this is likely to be challenging in our setting, where follow-up is already problematic.

The limitations of the study include the small sample size and inconsistent follow-up.

The high sensitivity with low specificity poses a danger of over-diagnosing at-risk infants, overburdening already overloaded high-risk clinics, and increasing parental anxiety.

## Conclusion

This study suggests that some abnormalities in subcategories of the MOS-R, such as absent FMs and abnormal finger variability, may predict suboptimal development at 18 months. A combination of GMA and HINE, alone or with HIE severity and 48 h aEEG, may be a useful screen to identify infants who do not require intensive follow-up. However, these findings need to be tested in a larger study.

## Data Availability

The raw data supporting the conclusions of this article will be made available by the authors, without undue reservation.
